# Supplementary material for: Genetic Suppressor Element 1 (GSE1) Promotes the Oncogenic and Recurrent Phenotypes of Castration-Resistant Prostate Cancer by Targeting Tumor-Associated Calcium Signal Transducer 2 (TACSTD2)
Source: Cancers (Basel). 2021 Aug 5;13(16):3959. doi: 10.3390/cancers13163959 (PMC8392851; doi:10.3390/cancers13163959)
Supplement: Supplementary file 1 [file cancers-13-03959-s001.zip › cancers-1306327-supplementary.pdf]

Supplementary Table S1. Molecular parameters of the GSE1-TACSTD2 complex

**GSE1 - TACSTD2 complex**		
# Receptor interface residue(s)	# Ligand interface residue(s):	# Receptor-ligand interface residue pair(s):
SER 93A 4.957	GLU 1174A 3.238	93A - 1202A 4.957
GLU 94A 4.897	SER 1177A 4.446	94A - 1188A 4.897
HIS 95A 0.997	LYS 1179A 2.185	94A - 1202A 4.941
ALA 96A 2.854	GLN 1180A 3.676	95A - 1188A 3.307
VAL 98A 4.143	ARG 1188A 3.307	95A - 1189A 3.244
ASP 101A 3.522	LEU 1189A 3.244	95A - 1202A 3.992
VAL 128A 4.128	GLN 1190A 4.227	95A - 1203A 0.997
ASN 129A 2.837	ALA 1191A 3.117	96A - 1202A 2.854
VAL 133A 2.712	GLU 1192A 3.569	96A - 1203A 3.438
ARG 134A 2.749	LEU 1193A 2.526	98A - 1203A 4.143
ARG 135A 2.860	ASP 1194A 4.293	98A - 1204A 4.742
THR 136A 4.142	LEU 1202A 2.854	101A - 1205A 4.048
ASP 137A 3.139	PRO 1203A 0.997	101A - 1207A 3.522
VAL 149A 4.811	ALA 1204A 4.742	128A - 1209A 4.128
ARG 159A 3.238	MET 1205A 3.847	129A - 1209A 2.837
GLU 227A 3.340	TRP 1207A 3.340	133A - 1209A 2.712
GLU 254A 4.053	PRO 1208A 1.740	134A - 1194A 4.558
ARG 255A 3.117	ARG 1209A 2.071	134A - 1209A 2.986
THR 256A 4.465	GLY 1210A 3.824	134A - 1210A 3.824
LEU 257A 3.214	TYR 1211A 2.749	134A - 1211A 2.749
ILE 258A 1.740	LEU 1212A 4.903	134A - 1212A 4.903
TYR 259A 2.185	LYS 1213A 4.765	134A - 1213A 4.765
TYR 260A 3.472		135A - 1209A 2.860
LEU 261A 4.906		136A - 1211A 4.142
ASP 262A 2.071		137A - 1211A 3.139
PRO 266A 3.855		149A - 1209A 4.811
PHE 268A 2.912		159A - 1174A 3.238
		227A - 1207A 3.340
		254A - 1189A 4.053
		255A - 1177A 4.446
		255A - 1189A 3.372
		255A - 1190A 4.227
		255A - 1191A 3.117
		256A - 1205A 4.465
		257A - 1179A 4.168
		257A - 1191A 3.214
		257A - 1193A 3.741
		257A - 1205A 3.847
		258A - 1193A 3.520
		258A - 1207A 4.126
		258A - 1208A 1.740
		259A - 1179A 2.185
		259A - 1180A 3.676
		259A - 1192A 3.569
		259A - 1193A 2.526
		259A - 1208A 3.564
		260A - 1207A 3.926
		260A - 1208A 3.472
		260A - 1209A 4.484
		261A - 1209A 4.906
		262A - 1209A 2.071
		266A - 1209A 3.855
		268A - 1194A 4.293
		268A - 1209A 4.532
		268A - 1211A 2.912
